# Porcine Circovirus (PCV) Genotype 2d-Based Virus-like Particles (VLPs) Induced Broad Cross-Neutralizing Antibodies against Diverse Genotypes and Provided Protection in Dual-Challenge Infection of a PCV2d Virus and a Type 1 Porcine Reproductive and Respiratory Syndrome Virus (PRRSV)

**DOI:** 10.3390/pathogens10091145

**Published:** 2021-09-06

**Authors:** Seok-Jin Kang, Sung-Min Bae, Hye-Jeong Lee, Young-Ju Jeong, Min-A Lee, Su-Hwa You, Hyang-Sim Lee, Bang-Hun Hyun, Nakhyung Lee, Sang-Ho Cha

**Affiliations:** 1 Division of Viral diseases, Animal and Plant Quarantine Agency, Gimcheon-si 39660, Korea; sj.kang75@korea.kr (S.-J.K.); maystem@korea.kr (H.-J.L.); ma5147@korea.kr (M.-A.L.); ysh0108@korea.kr (S.-H.Y.); leehs76@korea.kr (H.-S.L.); hyunbh@korea.kr (B.-H.H.); 2 KBNP, Anyang-si 14059, Korea; smbae@kbnp.co.kr (S.-M.B.); longred0212@hanmail.net (Y.-J.J.); nhlee21@kbnp.co.kr (N.L.); 3 Division of Foot-and-Mouth disease Research, Animal and Plant Quarantine Agency, Gimcheon-si 39660, Korea

**Keywords:** porcine circovirus type 2, recombinant VLP, vaccine, cross-neutralization

## Abstract

As PCV2d infection has been continuously reported in swine farms in which pigs were vaccinated with PCV2a- or 2d-based vaccines, we attempted to develop a novel vaccine using a PCV2d-based capsid to enhance its protective efficacy. In this study, recombinant virus-like particles (VLPs) of rPCV2a, rPCV2b and rPCV2d were synthesized from the capsid proteins of PCV2a, PCV2b and PCV2d field isolates, respectively. A cross-neutralization assay between the VLPs induced antisera and the field isolates demonstrated the broad cross-neutralizing activities of the rPCV2d-induced antisera. Then, the protective efficacy of rPCV2d as a vaccine candidate was investigated in commercial pigs by rPCV2d vaccination and a single- or dual-challenge infection using a PCV2d strain and a type 1 PRRSV strain. High levels of anti-PCV2d IgG and neutralizing antibodies were induced 3 weeks after vaccination. After the challenge infection, the average ADWG values of the vaccinated group were higher than those of the unvaccinated group. None or a significantly low amount of (*p* < 0.05) reduced PCV2 genomic DNA was found in the blood, saliva and tissues of the vaccinated pigs, when compared to the unvaccinated group. Moreover, macroscopic and microscopic lesions in the tissues were significantly (*p* < 0.05) reduced in the vaccinated groups. This study therefore suggests that rPCV2d may be highly useful for the control of diverse field genotypes.

## 1. Introduction

Porcine circovirus type 2 (PCV2) is the main etiologic agent for a multifactorial clinical disease (porcine circovirus-associated disease, PCVAD), causing major economic losses in the swine industry. In addition, the coinfection of PCV2 and other pathogens (PRRSV, PPV and *Mycoplasma hyopneumoniae*) increases the clinical severity of PCVAD, perhaps through synergistic effects, which may modulate PCV2 replication or clearance through an alteration of cytokine production [1,2,3].

According to recent publications, PCV2 is detected in approximately 87% of swine farms nationwide, and its major genotype is PCV2d [4]. In addition, the PCV2d genotype accounted for approximately 95% of PCV2 isolated in South Korea between 2016 and 2020 [5]. This genotype shift from PCV2a and 2b to PCV2d is occurring worldwide [4,6,7].

Most current commercial PCV2 vaccines are made using the PCV2a genotype capsid protein as an active ingredient. It has been reported that PCV2d infection in pigs is effectively cross-protected by current PCV2a vaccines [8,9]. However, PCV2 was not clearly removed from blood and tissues, even though these vaccines were able to decrease clinical symptoms. In our previous study, antiserum from PCV2a-immunized pigs showed different neutralization abilities against PCV2d isolates of different genetic backgrounds [5]. Inconsistent vaccine effectiveness against currently circulating PCV2 has recently increased in the field [10,11,12,13]. In addition, the residual viremia and viral load in the tissues of vaccinated pigs as a result of partial protection due to a low vaccine efficacy can cause serious economic losses in the swine industry through a reduced average daily weight gain and delayed shipments [14]. Taken together, this evidence suggests that the current PCV2a-based vaccines may have to be replaced in accordance with the genetic evolution of PCV2 field isolates.

The current PCV2 vaccines are made with either ORF2 capsid-based virus-like particles (VLPs) or inactivated whole viruses as active antigens, and are effectively used for controlling PCVAD. Considering the safety and efficacy of PCV2-VLPs, VLPs have recently been touted as next-generation subunit vaccine candidates. A VLP is a supramolecular particle resembling a native virus without its viral genome, which is formed following the assembly of monomeric structural proteins, expressed as recombinant proteins in the hosts [15,16]. As VLPs are noninfectious and able to elicit humoral and cell-mediated immune responses [17,18], VLP-based technology for vaccines is a good method for the design of foreign epitope carriers, and has been widely used in the manufacturing of both veterinary and human VLP vaccines [19,20,21].

In our preliminary data, it was reported that a PCV2d isolate (QIA244) was not neutralized by the antiserum derived from a PCV2a-based vaccination, whereas the antiserum raised by the QIA244 strain was able to effectively neutralize each PCV2a, 2b and 2d isolate according to an in vitro cross-neutralization assay. Therefore, in this study, we produced a PCV2d-VLP vaccine containing the recombinant protein encoding the ORF2 sequence of QIA244, and then evaluated its effectiveness in vitro and in vivo.

## 2. Results

### 2.1. Production of VLPs Expressed by Baculovirus Expression System

**Figure 1:**
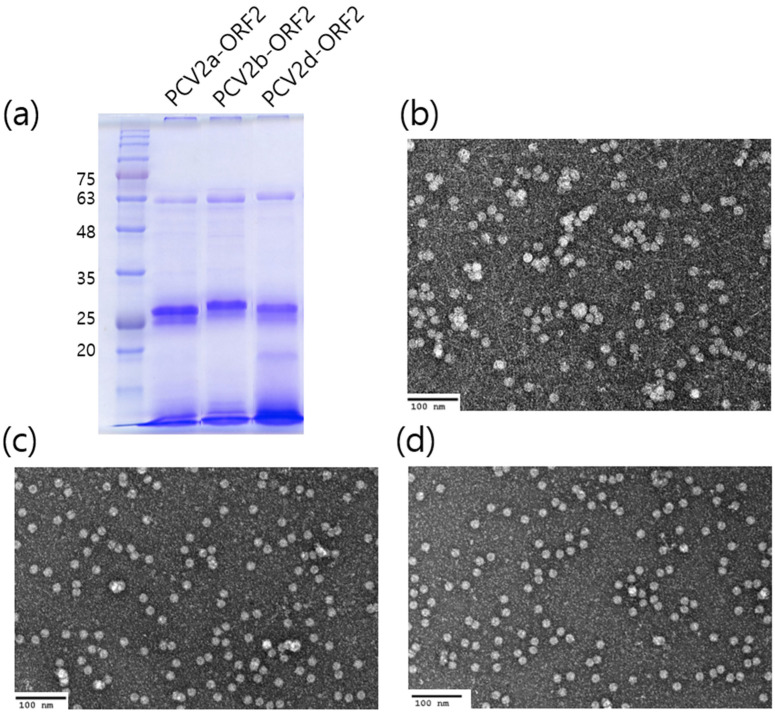
Characterization of the expressed recombinant PCV2-ORF2 protein. PCV2-ORF2 proteins were separated by 12 % SDS-PAGE (**a**). Assembled PCV2a VLPs (**b**), PCV2b-VLPs (**c**) and PCV2d-VLPs (**d**) were negatively stained and observed by a transmission electron microscopy (TEM). Scare bar is 100 nm.

The infection of Sf9 cells with the recombinant baculovirus strains, rAc-OP-PCV2a-ORF2, rAc-OP-PCV2b-ORF2 and rAc-OP-PCV2d-ORF2, resulted in the production of VLPs expressing PCV2a-ORF2, PCV2b-ORF2 and PCV2d-ORF2, respectively. The molecular mass of monomeric full-length PCV2a-ORF2, PCV2b-ORF2 and PCV2d-ORF2 proteins was approximately 27 kDa, shown as a dominant band in the SDS-PAGE gel of clarified cell lysate (Figure 1a) and confirmed by Western blotting analysis using a polyclonal anti-PCV2 antibody. PCV2a-ORF2, PCV2b-ORF2 and PCV2d-ORF2 proteins expressed in insect cells were self-assembled to form VLPs, and the purified PCV2-ORF2 protein was analyzed by TEM to verify the proper assembly of PCV2a-VLPs (Figure 1b), PCV2b-VLPs (Figure 1c) and PCV2d-VLPs (Figure 1d). The VLPs were approximately 17 nm in size and showed icosahedral symmetry.

### 2.2. Cross-Neutralization by VLP-Induced Antisera

In order to evaluate the cross-neutralization of VLP-induced antibodies, the antisera of mice and pigs immunized with VLPs of PCV2a (rPCV2a), 2b (rPCV2b) and 2d (rPCV2d) were cross-reacted with PCV2 isolates, including the PCV2a (QIA215), PCV2b (QIA418) and PCV2d genotypes (QIA169 and QIA244) (Table 1). Overall, the three rPCV2-induced antisera of mice and pigs showed significantly higher NA titers (1075 ± 400 to 6553 ± 1003 in mice and 1024 ± 512 to 5802 ± 2389 in pigs) against QIA215, QIA418 and QIA169 than those (58 ± 19 to 819 ± 307 in mice and 75 ± 28 to 725 ± 299 in pigs) against QIA244. Furthermore, there was no statistical difference in NA titers against QIA215, QIA418 and QIA169 among the three rPCV2-induced antisera, despite the average NA titer of the rPCV2d antisera being higher than that of other rPCV2 antisera. In terms of the neutralization between QIA244 and the three rPCV2-induced antisera, the NA titers (819 ± 307 in mice and 725 ± 299 in pigs) of the rPCV2d antisera against QIA244 were significantly higher than those of the rPCV2a antisera (64 ± 18 in mice and 75 ± 28 in pigs) and rPCV2b antisera (58 ± 19 in mice and 149 ± 56 in pigs) against QIA244.

### 2.3. Protection Efficacy of rPCV2d Vaccination in Pigs

**Figure 2:**
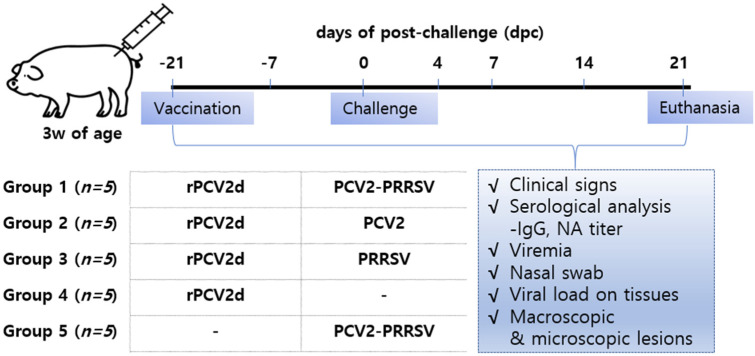
Experimental design. Five groups randomly assigned with 25 piglets: rPCV2d/PCV2-PRRSV (n = 5), rPCV2d/PCV2 (n = 5), rPCV2d/PRRSV (n = 5), rPCV2d/UnCh (n = 5) and UnVac/PCV2-PRRSV (n = 5). The piglets were vaccinated at −21 dpc (3 weeks of age), challenged at 0 dpc (6 weeks of age) and euthanized at 21 dpc (9 weeks of age). Clinical signs, serological analysis (total IgG and NA titer), viremia, nasal swab, viral load on tissues and pathological lesions were investigated during the period of experiments.

Pig study was designed as shown in Figure 2 and the detailed animal experiment described in Materials and Methods.

#### 2.3.1. Clinical Signs and ADWG

**Figure 3:**
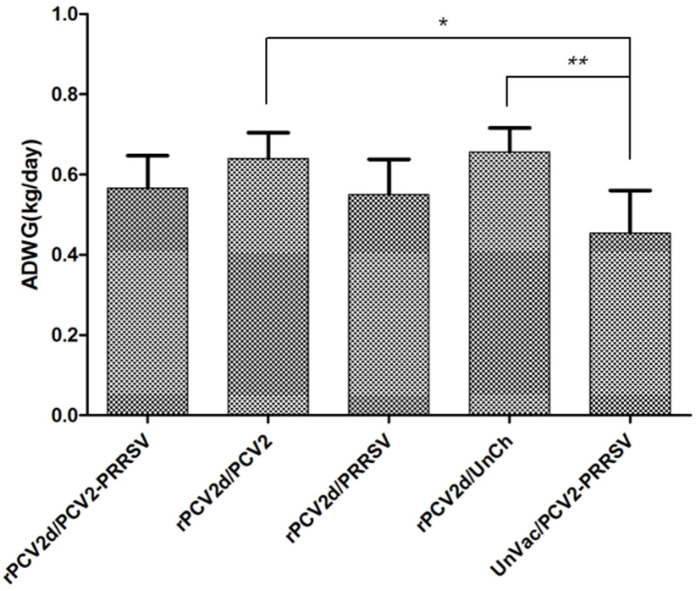
Average daily weight gain (ADWG). Body weight was estimated weekly until 21 dpc and the ADWG (kg/day) was calculated between 0 to 21 dpc. Significant difference was indicated by * *p* < 0.05 and ** *p* < 0.01.

Clinical signs, including a rectal temperature, diarrhea, depression and a rough haircoat, were not observed in any of the vaccinated groups for 21 days after the challenge infection, whereas the UnVac/PCV2/PRRSV group did show clinical signs. As shown in Figure 3, the ADWG of the UnVac/PCV2/PRRSV group (0.45 ± 0.11 kg/day) was slightly lower than that of the rPCV2d/PCV2/PRRSV (0.57 ± 0.08 kg/day) and rPCV2d/PRRSV groups (0.55 ± 0.09 kg/day), representing a PRRSV challenge, whereas it was significantly (*p* < 0.05) lower than that of the rPCV2d/PCV2 (0.64 ± 0.06 kg/day) and rPCV2d/UnCh groups (0.65 ± 0.06 kg/day) (Figure 3).

#### 2.3.2. Viral Load of PCV2 and PRRSV in Blood, Nasal Swab and Tissues

**Figure 4:**
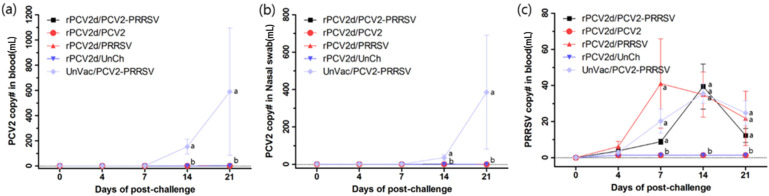
Quantification of PCV2 and PRRSV in blood and nasal swab. The PCV2 genomes were detected in blood (**a**) and nasal swab (**b**). The PRRSV genomes were also measured in blood (**c**). rPCV2d/PCV2-PRRSV (■), rPCV2d/PCV2 (●), rPCV2d/PRRSV (▲), rPCV2d/UnCh (▼) and UnVac/PCV2-PRRSV (◆). Different letters (a, b) indicate significant difference (*p* < 0.05) among groups.

In the blood and nasal swabs, the PCV2 genomes were detected in a significant amount (153 ± 45 to 590 ± 391 copies in blood and 37 ± 12 to 386 ± 236 copies in nasal swabs) for the UnVac/PCV2/PRRSV group from 14 to 21 dpc, whereas they were not detected or hardly detected in pigs of all vaccinated groups (Figure 4a,b). PRRSV genomes were detected only in PRRSV-challenged groups (rVac/PCV2/PRRSV, rVac/PRRSV and UnVac/PCV2/PRRSV) (Figure 4c). The amount of the PRRSV genome was increased from 7 dpc (9 ± 0.6 to 41 ± 5.2 copies) in blood, peaked at 14 dpc (35 ± 2.6 to 39 ± 3.2 copies) and rapidly dropped at 21 dpc (12 ± 1.5 to 25 ± 8.3 copies). In the tissues at 21 dpc, PCV2 genomes were limited or not detected in tissues in all of the rPCV2d-vaccinated groups (Table 2), whereas they were detected (5237 ± 3380 in lung, 19,720 ± 11,795 in tonsil, 11,172 ± 8150 in mesenteric LN and 3742 ± 2488 in inguinal LN) in the UnVac/PCV2/PRRSV group. PRRSV genomes were detected in the tissues (102 ± 12 to 155 ± 16 in lung, 117 ± 62 to 319 ± 48 in tonsil, 2.9 ± 0.0 to 26 ± 3.2 in mesenteric LN and 45 ± 2.8 to 113 ± 1.6 in inguinal LN) of all groups challenged with PRRSV.

#### 2.3.3. Serology of PCV2 and PRRSV

**Figure 5:**
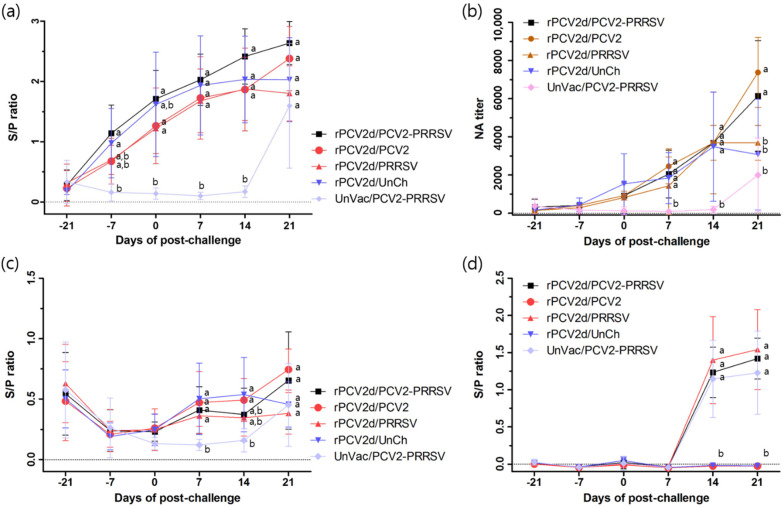
Serological analysis: (**a**) PCV2-specific antibody titers using an in-house ELISA based on PCV2d antigen; (**b**) serum neutralizing antibody titers; (**c**) PCV2-specific antibody titers using a PCV2a-based ELISA; (**d**) PRRSV-specific antibody titers. rPCV2d/PCV2-PRRSV (■), rPCV2d/PCV2 (●), rPCV2d/PRRSV (▲), rPCV2d/UnCh (▼) and UnVac/PCV2-PRRSV (◆). Different letters (a, b) indicate significant difference (*p* < 0.05) among groups.

Using an in-house ELISA assay coated with the PCV2d antigen, seroconversion was observed in the rPCV2d-vaccinated groups (rPCV2d/PCV2/PRRSV, rPCV2d/PCV2, rPCV2d/PRRSV and rPCV2d/UnCh) from −7 dpc (Figure 5a). The NA titers were significantly (*p* < 0.05) higher in the vaccinated groups (1434 ± 675 to 2458 ± 410) from 7 dpc than in the UnVac/PCV2/PRRSV group (93 ± 46) (Figure 5b). Furthermore, the VN titers were increased to 6144 ± 1295 and 7373 ± 819 at 21 dpc for the rPCV2d/PCV2/PRRSV and rPCV2d/PCV2 groups, respectively, whereas they were decreased from 14 dpc for the rPCV2d/PRRSV and rPCV2d/UnCh groups. The NA titer for the UnVac/PCV/PRRSV group increased to 1997 ± 866 at 21 dpc. Using the commercial ELISA assay, seroconversion was observed from 7 dpc, with a slight increase until 21 dpc for the rPCV2d/PCV2/PRRSV and rPCV2d/PCV2 groups (Figure 5c). On the other hand, the rPCV2d/PRRSV and rPCV2d/UnCh groups maintained nearly negative levels until 21 dpc, and the UnVac/PCV2/PRRSV group become seropositive at 21 dpc. Regarding PRRSV-specific antibodies, seroconversion was observed in all PRRSV-challenged groups from 14 dpc (Figure 5d).

#### 2.3.4. Gross and Histopathological Lesions

Macroscopic and microscopic lesions were scored, and the results are summarized in Table 3. The scores of macroscopic lesions (lung and inguinal LN) from the rPCV2d/PCV2/PRRSV (0.4 ± 0.6 and 0.0 ± 0.0), rPCV2d/PCV2 (0.2 ± 0.5 and 0.2 ± 0.5) and rPCV2d/UnCh (0.0 ± 0.0 and 0.0 ± 0.0) groups were significantly (*p* < 0.05) lower than those from the UnVac/PCV2/PRRSV group (1.2 ± 0.5 and 1.2 ± 0.5). The lesions were mainly observed in the lungs, such as peribronchial lymphocyte regeneration and interstitial pneumonia (Figure S1, Supplementary Materials). The scores of microscopic lesions (lung and inguinal LN) from the rPCV2d/PCV2/PRRSV (0.8 ± 0.5 and 0.0 ± 0.0), rPCV2d/PCV2 (0.0 ± 0.0 and 0.0 ± 0.0), rPCV2d/PRRSV (0.3 ± 0.5 and 0.0 ± 0.0) and rPCV2d/UnCh groups (0.0 ± 0.0 and 0.0 ± 0.0) were significantly lower (*p* < 0.05) than those from the UnVac/PCV2/PRRSV group (1.4 ± 0.6 and 0.8 ± 0.5). According to the histopathological analysis, the lesions were not detected in any of the vaccinated groups in the tonsils and both lymph nodes, whereas mild or moderate lymphocyte loss and macrophage proliferation were observed in the UnVac/PCV2/PRRSV group (Figure S2, Supplementary Materials). In terms of the macroscopic and microscopic scores of other tissues, there were no differences among groups.

## 3. Discussion

Despite extensive nationwide PCV2 vaccination, PCV2 has been actively circulating in swine farms, leading to continuous economic loss by inducing PCVAD in the field. Previous studies have reported that, even though commercial PCV2a-based vaccines are considered clinically protective against the PCV2d genotype [9,22], PCV2d strains were frequently isolated from clinically ill pigs in PCV2-vaccinated swine farms, possibly due to vaccine failure [7,12]. Recent genetic/antigenic characterization of the field isolates from PCVAD-affected farms revealed that over 90% of PCV2 was represented by the PCV2d genotype in the Republic of Korea, and the isolates showed low neutralizing cross-reactivity among genotypes or strains of PCV2 [5]. It was suggested that the low cross-neutralization activity may account for the active circulation of PCV2 and the occurrence of vaccine failure in vaccinated swine farms. Accordingly, cross-neutralization among the field isolates was investigated using mouse and pig antisera induced by rVLPs (rPCV2a, rPCV2b and rPCV2d) as an active ingredient of PCV2 vaccines. Among the tested VLPs, the PCV2d VLP showed broad cross-reactivity against several field isolates, and its protective efficacy against the PCV2d challenge infection was evaluated in pigs.

Regarding the recognition characteristics of antisera induced by the VLPs, NA titers of all antisera against the QIA244 strain were significantly lower than those against other viruses, whereas the neutralizing titer of antisera induced by rPCV2d was significantly higher than that induced by other VLPs for all field isolates, including QIA244. The QIA244-based VLP (rPCV2d) demonstrated a broad recognition of diverse genotypes or strains of PCV2, as importantly reflected in a previous study, which showed that antisera induced by the inactivated PCV2d genotype strain, QIA244, showed broad neutralizing activity [5]. Therefore, the use of rPCV2d as a vaccine candidate was expected to induce a better protective efficacy than the current PCV2a-based vaccines. On the other hand, it was unexpected that the neutralization antibody titer of rPCV2d antisera against QIA244 (819 ± 307 in mice and 725 ± 299 in pigs) would be significantly lower than other field isolates (ranged from 4915 ± 1389 to 6553 ± 1003 in mice and 2816 ± 1280 to 5802 ± 2389 in pigs), regardless of the genotype, which deserves attention in future studies.

For the evaluation of vaccination efficacy using rPCV2d, we first had to establish a dual-infection model of PCV2 and PRRSV in order to reproduce PCVAD in pigs under experimental conditions, since the induction of the typical manifestation of the PCV2-associated disease in commercial piglets delivered from sows vaccinated with the PCV2 vaccine requires a mixed infection of PRRSV, PPV and *Mycoplasma hyopneumoniae* [1,2,3]. In the relationship between PCV2 and PRRSV, PCV2 does not affect PRRSV replication or lesions, whereas PRRSV increases PCV2 DNA loads in the sera and tissues of coinfected pigs [23,24,25]. In the present study, we confirmed that the challenge with the PCV2 field isolate, in combination with type 1 PRRSV, induced a high viremia and viral load of PCV2 in tissues, as well as increased lung lesions, compared to a single-challenge with PCV2 or PRRSV. However, the pathogenicity of PRRSV was not affected by the coinfection of PCV2, confirming that the dual-infection of PCV2 and PRRSV can be used to appropriately evaluate the effectiveness of newly developed PCV2 vaccines.

Vaccination with rPCV2d induced a significantly high level of PCV2-specific IgG and neutralizing antibodies in pigs. For the evaluation of the PCV2d-specific IgG titer, an in-house ELISA analysis using PCV2d VLPs was developed to overcome the inaccurate immune assessment, which was possibly caused by the antigenic variability of VLPs (this study) and field isolates [5]. In comparison to the commercial ELISA kit, the in-house ELISA kit confirmed the obvious induction and chronological development of PCV2-specific IgG titers in the vaccinated pigs, whereas the commercial ELISA kit failed to detect the immune responses by the PCV2d VLP vaccination, as shown in Figure 5c. The negative effect of the ELISA antigenic difference suggests that the current commercial ELISA kit may have to be revalidated for diagnostic performance or newly developed in accordance with the antigen conformity of currently emerging genotypes.

In general, the alleviation of clinical signs and pathological lesions due to PCV2 infection is positively correlated with a reduction in viremia and viral load in tissues [9,22]. Since the PCV2 load in blood results in a poor growth performance, thus eventually delaying shipments [14], and since the levels of the NA titer show a good correlation with a reduction in PCV2 loads [26], stronger immune responses and broad recognition followed by a vaccination will be necessary for better effective protection against field isolates.

At 3 weeks after vaccination (0 dpc), the NA titer (819 ± 338 to 1536 ± 706) was not significantly different among vaccinated groups, whereas it showed a drastic increase in the vaccinated and challenged groups due to the anamnestic response following the challenge infection. Even if SN titers in the previous studies after vaccination [27,28] ranged from 1:32 to 1:64 within 42–75 days post vaccination, it may not be appropriate to suggest that SN titers induced by vaccination in this study were significantly higher than those in other studies, since the immunization level for PCV2 appears to be significantly different according to reagents and procedures used for the evaluation. However, as the SN titers on average, induced by CircoFlex (Boehringer Ingelheim, Ingelheim, Germany), one of the commercial vaccines, were 1:16 for QIA244 and 1:516-1024 for other genotypes PCV2s in the comparative study of the pig immunization capability of Table 1 (the data not shown), it was suggested that the developed vaccine may induce SN titers at a higher level than that of the commercial vaccine.

In the present study, vaccination with rPCV2d demonstrated a high level of protective capability against PCV2 infection. The rPCV2d vaccination significantly diminished the virus shedding and viral load of the challenged strain QIA244 in blood and nasal swabs, as proven in the little existence of PCV2 genomes at a similar level to the unchallenged group (rPCV2d/UnCh). Some previous studies reported that there was still a secretion of the virus in saliva and significant viremia, even in vaccinated animals [27,29]. Furthermore, vaccination with rPCV2d completely protected pigs from macroscopic or microscopic pathological lesions in the lung and inguinal LN. Interestingly, despite no obvious pathological lesions in mesenteric LNs and tonsils in the UnVac/PCV2/PRRSV group, PCV2 viral genomes in the tissues were detected at a higher level than those in the lung and inguinal LNs showing pathological lesions. This noncorrelation between the viral genome level and pathological lesion presence is inconsistent with previous studies [14,30] and should be further elucidated in the future. Regardless of the observation of pathological lesions, vaccination with rPCV2d successfully prevented viral loads in all tissues investigated in this study. Importantly, the average ADWG values of the vaccinated groups were higher than those of the unvaccinated group, implying that the vaccination of rPCV2d should be useful to protect livestock productivity against PCV2. Meanwhile, no statistical significance in the data analysis was observed among type 1 PRRSV-infected groups, indicating which group was affected by the virulence of PRRSV, as shown in similar levels of PRRSV viral loads in blood and tissues among PRRSV-infected groups. Thus, it was suggested that the virulence of PRRSV used in the dual-infection model should be more carefully controlled in the evaluation of the protection efficacy by the PCV2 vaccination. Collectively, this study demonstrated that rPCV2d has great potential to be used as a new vaccine to protect pigs from PCVAD caused by the currently prevalent strains of diverse PCV2 genotypes (PCV2a, PCV2b and PCV2d), with an enhanced capability of prevention.

## 4. Materials and Methods

### 4.1. Viruses and Cells

The field isolates of PCV2 were obtained from swine farms affected by PCVAD, as described in a previous study [5]. PCV2 field strains of QIA215 (PCV2a), QIA418 (PCV2b), QIA169 and QIA244 (PCV2d) were propagated in PCV-free PK15 cells cultured in DMEM supplemented with 5% FBS (Gibco-BRL, NY, USA) and 1% penicillin and streptomycin (Millipore, MA, USA). Type 1 PRRSV was isolated from piglets with respiratory problems and propagated in MARC-145 cells cultured in RPMI-1640 medium supplemented with 10% FBS and 1% penicillin and streptomycin.

PCV2d and type 1 PRRSV were titrated on PK15 and MARC-145 cells in quadruplicate, respectively. Briefly, serial dilutions (log_10_) of culture supernatants were exposed to each cell for 2 h, and then washed twice with 1× PBS. After 24 h, viral titers of PCV2 and PRRSV were determined by immunofluorescent staining with a PCV2-specific monoclonal antibody and according to the cytopathic effect, respectively, using the Spearman–Karber TCID_50_ method.

### 4.2. Production of Recombinant VLP Using Baculovirus Expression System (BES)

Sf9 cells were cultured in Erlenmeyer flasks (Corning, NY, USA) at 27 °C using Sf-900III serum-free medium (Invitrogen, Waltham, CA, USA) for the propagation of baculovirus. ORF2-based VLPs were produced in Sf9 cells using the BES. The full-length *ORF2* gene from the QIA244 strain, PCV2d genotype, was synthesized by optimizing the codons for expression in insect cells, before subcloning into a pOET1 transfer vector. To generate the recombinant baculovirus, Sf9 cells were co-transfected with the transfer vector plasmid and FlashBAC DNA (Oxford Expression Technologies, Oxford, UK). The resultant baculoviruses were designated as rAc-OP-PCV2d-ORF2. The virus was propagated by serial passages in Sf9 cells, and then harvested and titrated using a plaque assay. Additionally, rAc-OP-PCV2a-ORF2 and rAc-OP-PCV2b-ORF2, using the *ORF2* sequences of PCV2a (NCBI accession no. KF871067.1) and PCV2b (NCBI accession no. AY099500.1), respectively, were produced according to the same procedure. PCV2 VLPs were produced in Sf9 cells at a cell density of 1 × 10^6^ cells/mL with a multiplicity of infection (MOI) of 0.5, with respect to the recombinant baculoviruses, named rPCV2a, rPCV2b and rPCV2d as a function of the genotype used (ORF2a, ORF2b and ORF2d, respectively).

### 4.3. Analysis of PCV2 Virus-like Particles (VLPs)

After infection of Sf9 cells, the supernatant was separated by 12% SDS-PAGE. The quantity of PCV2 VLPs produced in Sf9 cells was measured by a densitometry scanner using ImageQuant TL software (GE healthcare, Chicago, IL, USA), before comparing with a bovine serum albumin (BSA) standard curve.

Transmission electron microscopy analyses were performed using traditional methods. Briefly, the PCV2 VLPs were adsorbed onto a carbon-coated copper grid for 3 min at room temperature (RT). The grid was negatively stained with 2% (*w*/*v*) aqueous uranyl acetate. Micrographs were recorded with a transmission electron microscope (Hitachi H7100FA, Tokyo, Japan).

### 4.4. Cross-Neutralization by rPCV2a, rPCV2b and rPCV2d-Induced Antisera

Regarding mouse immunization, 20 specific-pathogen-free (SPF) female BALB/c mice (6 weeks old) were purchased and reared in the animal housing facility. They were randomly allocated to four groups: rPCV2a (*n* = 5), rPCV2b (*n* = 5), rPCV2d group (*n* = 5) and nonimmunized group (*n* = 5). Mice were inoculated intraperitoneally with a 200 µL solution containing 20 µg of rPCV2a, rPCV2b and rPCV2d VLPs, along with a carbomer adjuvant. For pig immunization, 12 commercial crossbred piglets (3 weeks old) without PCV2 antigen and antibody were purchased and intramuscular immunized with a 1 mL solution containing 200 µg of rPCV2a (*n* = 3), rPCV2b (*n* = 3) and rPCV2d (*n* = 3), and no VLP (*n* = 3) as a negative control. An identical booster immunization was conducted at 2 weeks post-primary-immunization (wpi) in mice and pigs. At 4 wpi, serum samples were collected from the mice and pigs of all experimental groups to measure neutralizing antibody titers against PCV2a (QIA215), PCV2b (QIA418) and PCV2d (QIA169 and QIA244).

### 4.5. Protection Efficacy by rPCV2d Vaccination in Pigs

#### 4.5.1. Pig Studies

Commercial crossbred 3-week-old piglets free of *Mycoplasma* spp. and PRRSV, with either negative or low PCV2-specific IgG antibody titers without PCV2 circulation, were used. The protective efficacy of the recombinant vaccine (rPCV2d) was evaluated by a dual-challenge of PCV2 and PRRSV. As shown in Figure 2, 25 pigs were randomly divided into five groups (five pigs each group), vaccinated at 3 weeks old and challenged at 6 weeks old (Figure 2). Among the four groups vaccinated with rPCV2d, the first group (rPCV2d/PCV2/PRRSV) was challenged with PCV2 and PRRSV, the second group (rPCV2d/PCV2) was challenged with PCV2, the third group (rPCV2d/PRRSV) was challenged with PRRSV and the fourth group (rPCV2d/UnCh) remained unchallenged. The remaining group (UnVac/PCV2/PRRSV) was unvaccinated and challenged with PCV2 and PRRSV. Among the challenge viruses, PCV2 was represented by QIA244 as the PCV2d genotype with a homologous ORF2 sequence with rPCV2d, whereas PRRSV was a type I PRRSV field isolate with medium to low pathogenicity in our preliminary study (data not shown). The titer of the two challenge viruses was 10^5^ TCID_50_/mL. Each piglet was intranasally injected with 6 mL of PCV2 (3 mL)/PRRSV (3 mL) for dual-infection and 6 mL of PCV2 (3 mL)/PBS (3 mL) or 6 mL of PRRSV (3 mL)/PBS (3 mL) for single-infection. All pigs were euthanized at 21 days post-challenge (dpc) by an intravenous injection of sodium pentobarbital and electrocution. The methods associated with the studies were approved by the KBNP Institutional Animal Care and Use Committee.

#### 4.5.2. Clinical Signs and Average Daily Weight Gain (ADWG)

After the challenge, the clinical signs of all pigs were observed daily, including rectal temperature, presence of respiratory or digestive disorders, coughing and lameness. Body weight was measured weekly until 21 dpc, and the ADWG (kg/day) was calculated using body weights between 0 and 21 dpc.

#### 4.5.3. Quantification of PCV2 and PRRSV

Total nucleic acid (DNA and RNA) extraction of PCV2 and PRRSV was performed using a commercial kit (DNeasy Blood & Tissue Kit, Qiagen, Hilden, Germany), followed by quantitative RT-PCR using the LightCycler 480 II (Roche Diagnostics, Mannheim, Germany) to quantify the genomic copy numbers in serum, nasal swabs and tissues. The genome copies of PCV2 in the samples were detected using TB Green^TM^ Premix Ex Taq^TM^ (TaKaRa, Kyoto, Japan), with a specific primer set for the *ORF2* gene of PCV2: forward primer 5′–CACGGATATTGTAGTCCTGGTCG–3′ and reverse primer 5′–CGCACCTTCGGATATACT–3′. The PCR was performed according to the following conditions: denaturation at 95 °C for 5 s, followed by annealing at 64 °C for 10 s for up to 45 cycles. The genome copies of PRRSV were detected using the QuantiTect Probe PCR Kit (Qiagen, Hilden, Germany) with the *ORF7*-specific primers as follows: forward primer 5′–GTACAATGATAAAGTCCCAGCAC–3′, reverse primer 5′–GAATCAAGCGCACTGTATGAGC–3′ and probe primer 5′–CCTCTGCTTGCAATCGATCCAGAC–3′. 

After obtaining standard curves for the primers, amplification of the target gene was carried out. The melting curves of amplified products were analyzed to verify the specificity of the PCR, considering positive samples as those with a cycle threshold (Ct) <35 cycles.

#### 4.5.4. ELISA Analysis

The level of serum antibody against PCV2 and PRRSV was measured using commercial ELISA kits as follows: PCV2 (VDpro PCV2 Ab kit, Median diagnostics, Seoul, Korea) and PRRSV (IDEXX PRRS X3 Ab test, IDEXX Laboratories Inc. Westbrook, ME, USA). The optical density (OD) of PCV2 and PRRSV was read at 450 nm and 650 nm, respectively, according to the manufacturer’s instructions. Serum samples were considered positive for PCV2 and PRRSV if the sample-to-positive (S/P) ratio was >0.4.

For the PCV2d in-house ELISA, all samples were analyzed as described by Blanchard and colleagues [31]. Briefly, 96-well plates were coated with the purified PCV2d capsid protein and blocked with 1% BSA in PBS. Subsequently, 100 µL/well of the diluted serum at 1:1600 in PBS was added and incubated at RT for 30 min. Following three washing steps with PBS containing 0.5% Tween-20 (PBS-T), the final dilution of the horseradish peroxidase-labeled goat anti-pig IgG antibody (Abcam, Cambridge, MA, USA) was added and then further incubated at RT for 30 min. The wells were washed three times, and the reaction was developed using 100 µL of tetramethyl benzidine solution (Thermo, Waltham, MA, USA). After incubation for 15 min at RT, the OD was read at 450 nm.

#### 4.5.5. Viral Neutralization Assay

The fluorescent focus neutralization (FFN) test was performed using each strain of PCV2 as described by Meerts and colleagues [32]. Briefly, 200 TCID_50_ PCV2 at a volume of 100 µL was incubated for 1 h at 37 °C with 100 µL serial dilutions of antisera collected from experimental groups. After incubation, this mixture was added to 5 × 10^3^ PK15 cells in four wells of a 96-well plate. After 2 h at 37 °C, the cultured cells were washed twice in 1× PBS, and fresh medium was added. At 3 dpi, cells were fixed in 80% acetone (Merck Millipore, MA, USA) in distilled water. PCV2-infected PK15 cells were stained as previously described [5]. The neutralizing antibody (NA) titer was calculated using the 90% virus neutralization test (VNT90), defined as the highest serum dilution that protects more than 90% of cells from PCV2 infection.

#### 4.5.6. Gross Pathology and Histopathology

All pigs were humanely euthanized by an intravenous injection of sodium pentobarbital and electrocution at 9 weeks of age (21 dpc). Macroscopic and microscopic lesions were analyzed morphometrically as previously described [33]. Macroscopic lesions of the lung, tonsil, mesenteric LN and inguinal LN were scored as 0 (normal), 1 (mild), 2 (moderate) or 3 (severe) in a blinded fashion. Sections of the lung, tonsil, mesenteric LN and inguinal LN were collected at necropsy, fixed in 10% neutral buffered formalin and processed routinely for histological examination. Microscopic lesions were evaluated by a veterinary pathologist blinded to the treatment groups. Sections of the lung were scored for the presence and severity of interstitial pneumonia, scored as 0 (no lesions), 1 (mild interstitial pneumonia), 2 (moderate interstitial pneumonia) or 3 (severe interstitial pneumonia). Lymphoid tissues, including tonsil and lymph nodes, were evaluated for the presence of lymphoid depletion, scored as 0 (normal), 1 (mild), 2 (moderate) or 3 (severe).

### 4.6. Statistical Analysis

The results were expressed as the mean ± standard error (SE) for triplicate experiments (*n* = 3). The statistical significance was determined using GraphPad Prism 7 software (GraphPad Software, Inc., San Diego, CA, USA) with one-way analysis of variance (ANOVA) and Dunnett’s multiple comparisons test to compare groups. A *p*-value < 0.05 was considered to be statistically significant.

## Figures and Tables

**Table 1:** Neutralizing antibody titers (mean ± SE) of VLPs (rPCV2a, rPCV2b and rPCV2d) in mice (n = 5) and pigs (n = 3).

PCV2 Isolate (Genotype)	Anti-Mouse Sera			Anti-Pig Sera		
	rPCV2a	rPCV2b	rPCV2d	rPCV2a	rPCV2b	rPCV2d
QIA215 (2a)	2867 ± 1351	2509 ± 1449	4915 ± 1389	2047 ± 1023	2389 ± 903	5632 ± 2560
QIA418 (2b)	4506 ± 1505	2688 ± 1422	6553 ± 1003	4096 ± 2048	4437 ± 2076	5802 ± 2389
QIA169 (2d)	2150 ± 570	1075 ± 400	5325 ± 1229	1024 ± 512	3413 ± 683	2816 ± 1280
QIA244 (2d)	64 ± 18	58 ± 19	819 ± 307	75 ± 28	149 ± 56	725 ± 299

**Table 2:** Viral load (mean ± SE) of PCV2 and PRRSV in lung, tonsil, mesenteric LN and inguinal LN.

Groups	PCV2 Genomic DNA (per 200 ng Total DNA)				PRRSV Genomic RNA (per 200 ng Total RNA)			
	Lung	Tonsil	Mesenteric LN	Inguinal LN	Lung	Tonsil	MesentericLN	Inguinal LN
1	5.1 ± 1.1 ^a^	2.8 ± 0.6 ^a^	0.5 ± 0.3 ^a^	1.3 ± 0.9 ^a^	155.3 ± 16.1 ^a^	117.2 ± 62.0 ^a^	5.5 ± 0.0 ^a^	44.8 ± 2.8 ^a^
2	6.0 ± 1.6 ^a^	4.2 ± 0.6 ^a^	0.4 ± 0.1 ^a^	1.4 ± 0.8 ^a^	0.0 ± 0.0 ^b^	0.0 ± 0.0 ^b^	0.0 ± 0.0 ^b^	0.0 ± 0.0 ^b^
3	1.5 ± 0.1 ^a^	1.6 ± 0.2 ^a^	0.1 ± 0.0 ^a^	0.1 ± 0.0 ^a^	101.6 ± 11.4 ^c^	319.1 ± 48.2 ^c^	2.9 ± 0.0 ^a^	113.4 ± 1.6 ^c^
4	1.3 ± 0.1 ^a^	2.1 ± 0.7 ^a^	0.8 ± 0.7 ^a^	0.1 ± 0.0 ^a^	0.0 ± 0.0 ^b^	0.0 ± 0.0 ^b^	0.0 ± 0.0 ^b^	0.0 ± 0.0 ^b^
5	5237 ± 3380 ^b^	19,720 ± 11,795 ^b^	11,172 ± 8150 ^b^	3742 ± 2488 ^b^	106.9 ± 14.1 ^c^	171.4 ± 16.4 ^a^	25.8 ± 3.2 ^c^	48.9 ± 7.4 ^a^

Different letters (a, b, c) indicate that the groups are significantly (*p* < 0.05) different from each other.

**Table 3:** Scores (mean ± SE) of macroscopic and microscopic lesions in lung, tonsil, mesenteric LN and inguinal LN.

Groups	Macroscopic Lesions				Microscopic Lesions			
	Lung	Tonsil	Mesenteric LN	Inguinal LN	Lung	Tonsil	Mesenteric LN	Inguinal LN
1	0.4 ± 0.6 ^a,c^	0.0 ± 0.0	0.0 ± 0.0	0.0 ± 0.0 ^a^	0.8 ± 0.5 ^a^	0.0 ± 0.0	0.0 ± 0.0	0.0 ± 0.0 ^a^
2	0.2 ± 0.5 ^a^	0.0 ± 0.0	0.2 ± 0.5	0.2 ± 0.5 ^a^	0.0 ± 0.0 ^b^	0.0 ± 0.0	0.0 ± 0.0	0.0 ± 0.0 ^a^
3	1.0 ± 0.0 ^b,c^	0.0 ± 0.0	0.0 ± 0.0	1.0 ± 0.0 ^b^	0.3 ± 0.5 ^a,b^	0.0 ± 0.0	0.0 ± 0.0	0.0 ± 0.0 ^a^
4	0.0 ± 0.0 ^a^	0.0 ± 0.0	0.2 ± 0.5	0.0 ± 0.0 ^a^	0.0 ± 0.0 ^b^	0.0 ± 0.0	0.0 ± 0.0	0.0 ± 0.0 ^a^
5	1.2 ± 0.5 ^b^	0.0 ± 0.0	0.4 ± 0.6	1.2 ± 0.5 ^b^	1.4 ± 0.6 ^c^	0.6 ± 0.9	0.4 ± 0.6	0.8 ± 0.5 ^b^

Different letters (a, b, c) indicate significant (*p* < 0.05) difference among groups.

## Data Availability

All relevant data are provided within the article and supplementary materials.

## Supplementary Materials

The following are available online at https://www.mdpi.com/article/10.3390/pathogens10091145/s1: Figure S1, macroscopic observation of lung lesions; Figure S2, microscopic observation of lesions in lung, tonsil, mesenteric and inguinal LN.
