# Supplementary material for: Porcine Circovirus (PCV) Genotype 2d-Based Virus-like Particles (VLPs) Induced Broad Cross-Neutralizing Antibodies against Diverse Genotypes and Provided Protection in Dual-Challenge Infection of a PCV2d Virus and a Type 1 Porcine Reproductive and Respiratory Syndrome Virus (PRRSV)
Source: Pathogens. 2021 Sep 6;10(9):1145. doi: 10.3390/pathogens10091145 (PMC8464671; doi:10.3390/pathogens10091145)

Supplementary Materials

Figure S1. Macroscopic observation of lung lesions.

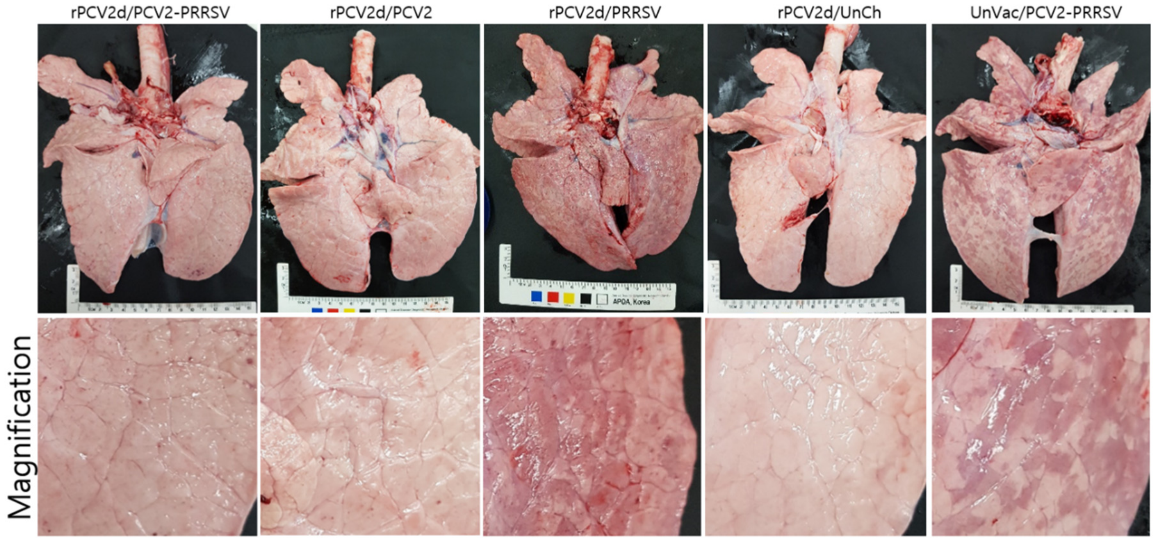

Figure S2. Microscopic observation of lesions in lung, tonsil, mesenteric and inguinal LN.

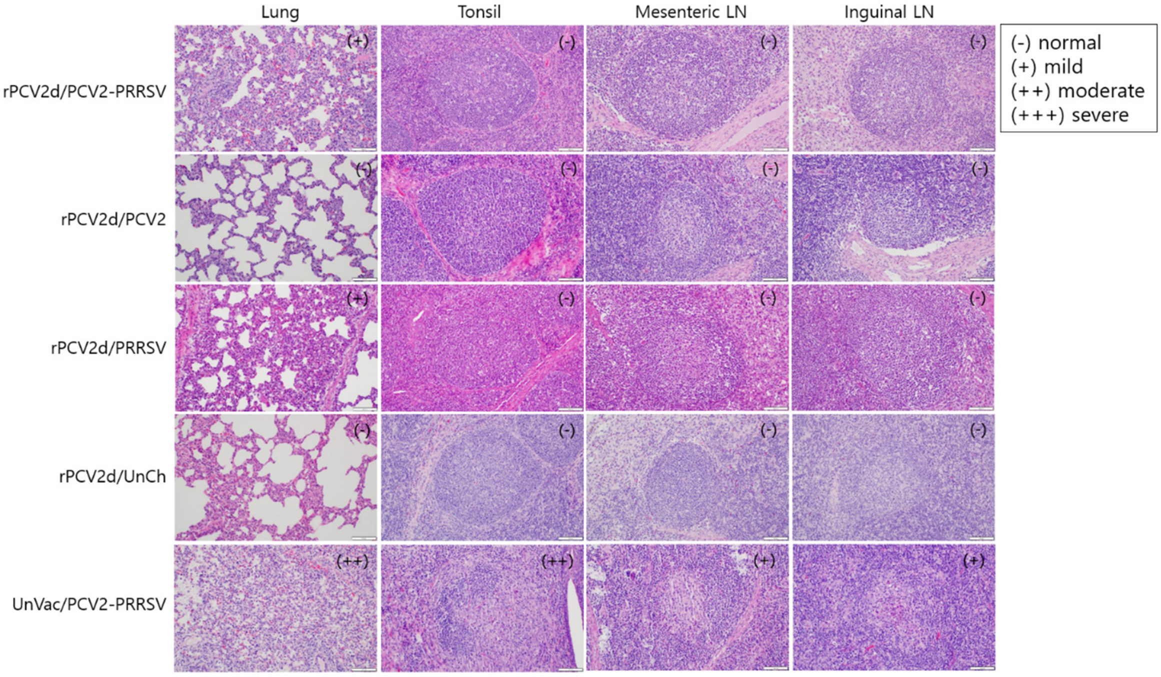

Supplement: Supplementary file 1 [file pathogens-10-01145-s001.zip › pathogens-1335177-supplementary.pdf]
